# Mass Die‐Off Events in Swarming Hyperiid Amphipods: Potential Drivers

**DOI:** 10.1002/ece3.70949

**Published:** 2025-02-01

**Authors:** Tamar Guy‐Haim, Anastasiia Iakovleva, Viviana Farstey, Ayah Lazar, Khristina Ermak, Arseniy R. Morov

**Affiliations:** ^1^ National Institute of Oceanography, Israel Oceanographic and Limnological Research Haifa Israel; ^2^ Department of Life Sciences Ben‐Gurion University of the Negev Beer Sheva Israel; ^3^ Department of Maritime Civilizations University of Haifa Haifa Israel; ^4^ The Interuniversity Institute for Marine Sciences Eilat Israel

**Keywords:** amphipod, mass mortality, mesoscale eddy, stranding, swarming behavior, zooplankton

## Abstract

Beach mass stranding events of marine organisms, widely documented worldwide, are triggered by a range of biotic and abiotic environmental factors, often unexplained. Such occurrences among pelagic crustaceans are less frequent, yet not uncommon. Here we studied mass mortality events of hyperiid amphipods—abundant members of pelagic zooplankton, commonly associated with gelatinous organisms. Our study examined consecutive mass die‐off and stranding events of free‐living hyperiids in the Red Sea during 2023 and 2024. We investigated three potential causes: semelparous reproduction, thermal stress, and physical oceanographic conditions. To place our findings in a broader context, we further performed a global review of hyperiid swarming and mass mortality events from scientific literature and a citizen science repository. Morphological and molecular analyses confirmed that the hyperiid species in the die‐off events at the Red Sea was *Anchylomera blossevillei* (Phrosinidae). The balanced male: female sex ratio (0.99), combined with the absence of gravid or brooding females, led to the rejection of semelparity as a driving factor. The environmental data did not indicate thermally stressful conditions, and no evidence of parasitic infection was found. Nonetheless, previous studies have shown that under weak wind conditions, as measured during the stranding events, coherent cyclonic eddies with diameters of 5–6 km are developed in the northern Gulf of Aqaba, persisting for about a day. These eddies can exceed velocities of 100 cm s^−1^ and may have facilitated the hyperiid stranding events. Future research should unveil the impacts of such events on marine ecosystems.

## Graphical abstract

We investigated mass mortality events of hyperiid amphipods, *Anchylomera blossevillei* , in the Red Sea during 2023 and 2024. Morphological and molecular analyses ruled out semelparity and thermal stress as causes, with evidence pointing to cyclonic eddies as a potential driver. A global review of hyperiid mass mortality events provided a broader context for our findings.

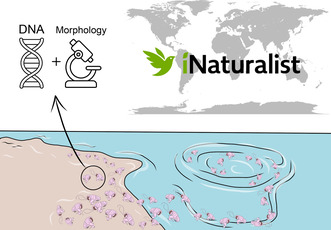

## 1 Introduction

Mass mortality and stranding of pelagic organisms are well documented worldwide, notably in cetaceans (Clarke et al. 2021), sea turtles (Vélez‐Rubio et al. 2022), fish (Hernández‐Miranda et al. 2010), jellyfish, and other cnidarians (Flux 2008; Baliarsingh et al. 2023). Natural and anthropogenic drivers of mass die‐offs and the consequent stranding events include infectious diseases (Arbelo et al. 2013), chemical or algal toxin exposure (Karlson et al. 2021), upwelling (Davenport 1995), abnormal weather and climatic conditions (Baliarsingh et al. 2023), bycatch (Wright et al. 2013), high‐intensity acoustic inputs (Cox et al. 2005), and semelparous reproductive strategy (Johnston et al. 2020). Stranding reports of pelagic crustaceans are less frequent, yet not rare, covering diverse taxa of decapods (Boyd 1967; Diez et al. 2012; Cimino et al. 2021), euphausiids (O'Brien, Ritz, and Kirkwood 1986; Hanamura, Saito, and Hayashi 2003), stomatopods (Romanov et al. 2015), and amphipods (Gray and McHardy 1967; Quigley et al. 2015; Brown and Gibbons 2022).

Amphipods of the suborder Hyperiidea are exclusively marine and strictly pelagic and are considered the third most abundant group in pelagic zooplankton communities, after copepods and krill (Bowman and Gruner 1973). They are commonly epipelagic to upper mesopelagic (Steinberg et al. 2008; Burridge et al. 2017; Espinosa‐Leal, Bode, and Escribano 2020), with an increasing number of species recorded from the deep sea (Gasca 2009; Lindsay and Pages 2010; Gasca and Haddock 2016). Hyperiids are often associated with gelatinous zooplankton, either as parasites, obligate commensals, or predators of tunicates, medusae, ctenophores, siphonophores, heteropod and pteropod mollusks, and radiolarians (Harbison, Biggs, and Madin 1977; Laval 1980; Phleger et al. 1999; de Lima and Valentin 2001; Gasca and Haddock 2004; Gasca, Hoover, and Haddock 2015).

Semelparity, characterized by a single reproductive episode followed by death, is a common reproductive strategy in amphipods (Varpe and Ejsmond 2018), including hyperiids (Percy 1993), and was previously used to explain hyperiid mass mortality events (Dunbar 1957; Wing 1976). Tornado‐shaped monospecific swarms of hyperiid amphipods, followed by beach stranding, were observed along the False Bay coastline in South Africa (Brown and Gibbons 2022). Although the majority of the individuals were females, semelparity was deemed to be an unlikely driver of the hyperiid mortality as only a low percentage of the females were gravid (bearing eggs in a brood pouch) (Brown and Gibbons 2022). Similar hyperiid swarms were previously documented in the water column in Keauhou Bay, Hawaii, followed by beach stranding (Lobel and Randall 1986). These swarms were attributed to a cold‐core cyclonic eddy that dominated the regional offshore mesoscale current flow field (Lobel and Robinson 1986).

Here we report mass mortality and stranding events of the hyperiid amphipod *Anchylomera blossevillei* (Phronimoidea, Phrosinidae) in the northern Gulf of Aqaba, Red Sea, which occurred during March and April 2023 and April 2024. Recently, marine heatwaves have been associated with mass mortality of reef fish in the Gulf of Aqaba (Genin et al. 2020). The high rate of the warming events at their onsets triggered pathogen infection, leading to fish mortality. Fungal pathogens were found in hyperiid amphipods (Ohtsuka et al. 2004; Bojko and Ovcharenko 2019); however, they had mostly an impact on reproduction rather than survival. Thus, we can hypothesize that the root cause for the successive annual hyperiid mass mortality events in the Gulf of Aqaba could be: (1) semelparous reproduction, (2) thermal stress, or (3) physical oceanographic conditions. We used integrative taxonomy to identify the hyperiid species and determined the sex ratio in the stranded assemblages to test semelparity as a potential root cause for the mass mortality. Potential thermal stress was tested using in situ temperature data from a nearby monitoring station, and the current regime was investigated using climatic data and previous research. We further present the global evidence of hyperiid blooms and die‐offs, using data gathered from the scientific literature and a citizen science repository, and discuss their potential drivers and ecosystem implications.

## 2 Materials and Methods

### 2.1 Sampling Site and Sample Collection

**FIGURE 1:**
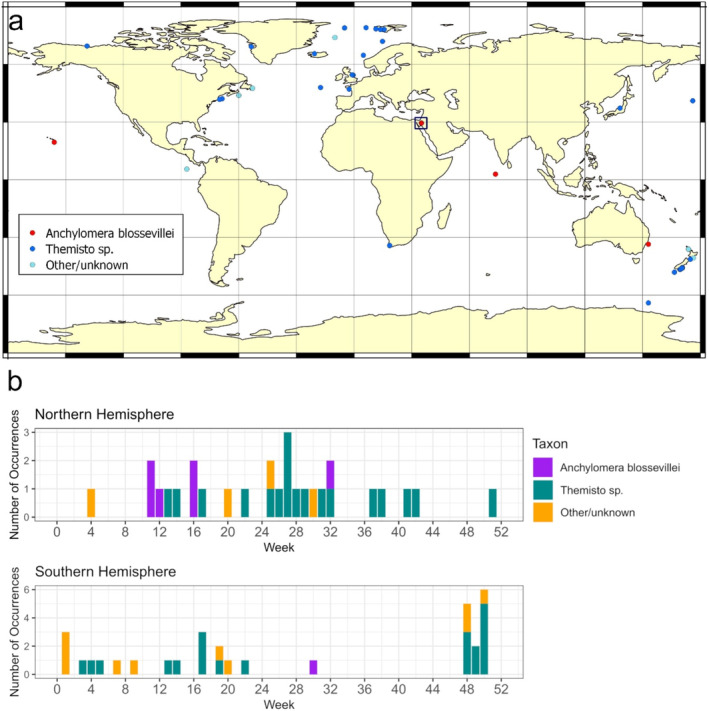
(a) Worldwide swarming and mass stranding reports of hyperiid amphipods based on literature search (ISI Web Of Science) and iNaturalist reports (accessed on 4 August 2024). Observations of *Anchylomera blossevillei* are presented in red circles, *Themisto* spp. in blue circles, and other/unidentified hyperiid taxa in light blue circles. The study site is indicated by a black square. (b) Weekly occurrences of swarming and mass stranding events of hyperiid amphipods in the northern and southern hemispheres. The full list of reports and references is detailed in Table 1.

**FIGURE 2:**
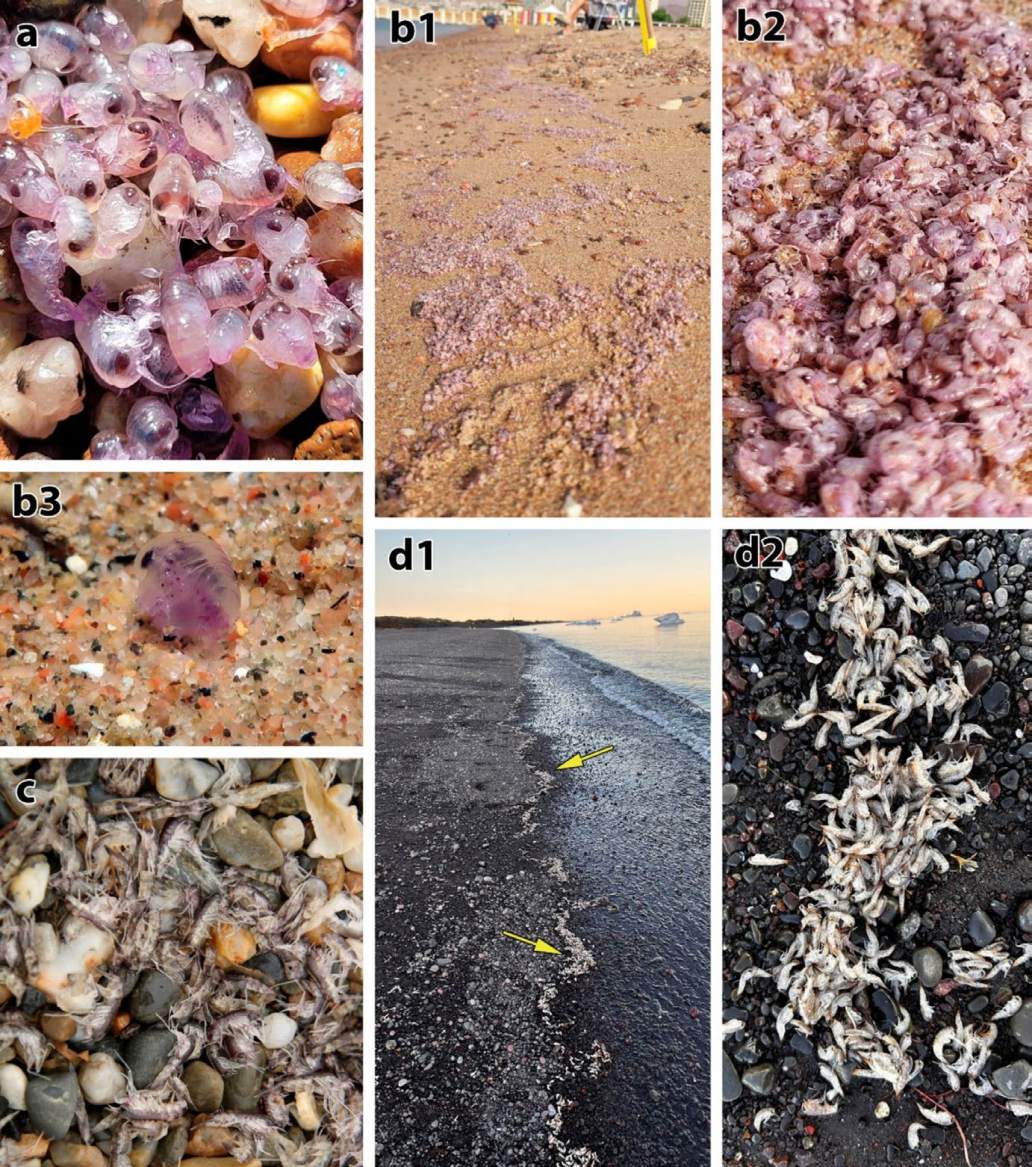
Mass die‐off and stranding of hyperiid amphipods. (a) *Anchylomera blossevillei* , Electric Company Beach, Eilat, 17 March 2023. (b1, b2) *A. blossevillei* , North Beach, Eilat, 23 April 2024. (b3) A frail individual of *A. blossevillei* underwater, North Beach, Eilat, 28 April 2024. (c) *Themisto libellula* , Prince Charles Foreland, Svalbard, 16 July 2020. (d1, d2) *T. libellula* , Disko Island, Greenland, 5 August 2023. Yellow arrows indicate line of stranded amphipods.

On March 17, 2023, a mass stranding event of hyperiid amphipods was reported by the Israel National Parks Authority (INPA) at the Electric Company Beach, Eilat, in the northern Gulf of Aqaba, Red Sea (29.5454° N, 34.9471° E, Figures 1a and 2a). The stranded carcasses were photographed, and ca. 500 individuals were collected. A month later, on April 22, 2023, similar reports were made by observers in North Beach, Eilat (29.5454° N, 34.9714° E).

On April 23, 2024, a mass stranding event of hyperiid amphipods was reported by INPA in the North Beach, Eilat (Figures 1a and 2b1,b2). Reports of “weak” amphipods in the shallow waters (Figure 2b3) and further mass accumulations of amphipod carcasses were made from April 23 to 28. The stranded carcasses were photographed, and ca. 500 individuals were collected.

From each sampling event, a subsample (30–40 individuals) was fixed in 70% ethanol, and the remaining sample was stored at −20°C pending analysis.

Surface temperature, wind speed, and wind direction were obtained from the National Monitoring Program at the Gulf of Eilat (https://iui‐eilat.ac.il/Research/NMPMeteoData.aspx). Temperature was recorded by a CTD (Sea‐Bird Electronics, USA), and the wind by a marine wind monitor (Young 05106, RM Young, USA).

### 2.2 Morphological Identification and Male: Female Ratio

In the lab at the National Institute of Oceanography, Israel Oceanographic and Limnological Research (IOLR), the ethanol‐fixed and thawed samples were examined under a stereomicroscope (SZX16, Olympus, Japan). Amphipod species and sex ratio were determined following the sexual dimorphism characters described in Milne‐Edwards (1830), Bowman and Gruner (1973), and Vinogradov et al. (1996). The presence of gregarine and ciliate infection was examined in the body cavities of the specimens following Prokopowicz et al. (2010).

### 2.3 Molecular Analysis

Ethanol‐preserved specimens from March 2023 and April 2024 were analyzed. Total genomic DNA was extracted from individual specimens using the DNeasy Blood and Tissue kit (QIAGEN, Germany) according to the manufacturer's specifications. Following DNA extraction, the 18S ribosomal gene (18S rRNA) was amplified using PCR with the universal primers #3F (5′‐GYGGTGCATGGCCGTTSKTRGTT‐3′) (Machida and Knowlton 2012) and 9R (5′‐GATCCTTCCGCAGGTTCACCTAC‐3′) (Giribet et al. 1996). Reaction conditions were as follows: 94°C for 2 min, followed by 35 cycles of 94°C for 15 s, 49°C for 30 s, and 72°C for 1 min, and a final elongation step of 72°C for 7 min. Obtained PCR products were separated on 1.5% agarose gel and stained with GelRed (Biotium Inc., USA). Purification and Sanger sequencing of the PCR products were performed by Hy Laboratories Ltd. (Rehovot, Israel). Sequence alignment was conducted using ClustalW embedded in MEGA v11.0 (Tamura, Stecher, and Kumar 2021). The best‐fitting substitution model was selected according to the Bayesian Information Criterion using maximum‐likelihood (ML) model selection in MEGA. ML analysis was performed using the K2 + G model with 1000 bootstrapping replicates.

### 2.4 Literature Review of Hyperiid Mass Mortality Events

In addition to the samples collected from the northern Gulf of Aqaba, further reporting of mass stranding events of hyperiid amphipods was obtained from the literature on 4 August 2024 using the following search in ISI Web of Science: (hyperiid*) AND (swarm OR bloom OR strand OR mortalit* OR die? off), and from the citizen science network iNaturalist (https://www.inaturalist.org/) based on photographic evidence in the category “Hyperiidea”.

## 3 Results

### 3.1 Environmental Conditions During Die‐Off and Stranding Events

**FIGURE 3:**
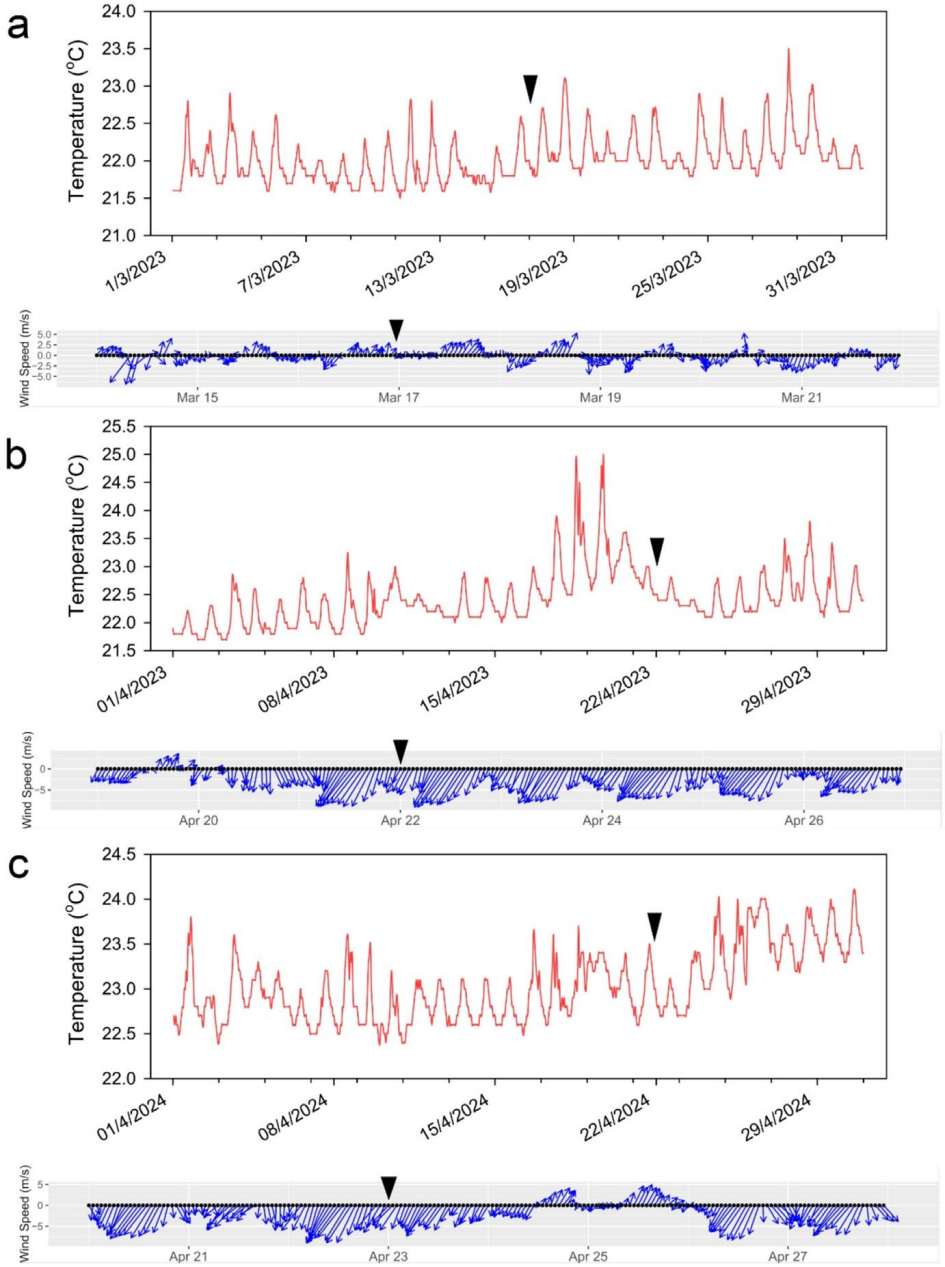
Water temperature, wind speed, and direction during hyperiid stranding events in the northern Gulf of Aqaba Eilat. The occurrences are marked by arrowheads. (a) March 17, 2023. (b) April 22, 2023. (c) April 23, 2024.

Mean (±standard deviation) daily surface temperature levels during the stranding events in the northern Gulf of Aqaba, Eilat, were 22.14°C ± 0.31°C, 22.48°C ± 0.14°C, and 23.05°C°C ± 0.29°C on March 17, 2023, April 22, 2023, and April 23, 2024, respectively. Wind speed and direction were 1.90 ± 1.52 m/s SSW, 6.95 ± 2.84 m/s NNE, and 1.75 ± 1.51 m/s NNE during March 17, 2023, April 22, 2023, and April 23, 2024, respectively. The moon phases varied during the stranding events and were waxing, new moon, and full moon (illumination of 28%, 4%, and 99%) during March 17, 2023, April 22, 2023, and April 23, 2024, respectively (Figure 3).

### 3.2 Morphological Description

**FIGURE 4:**
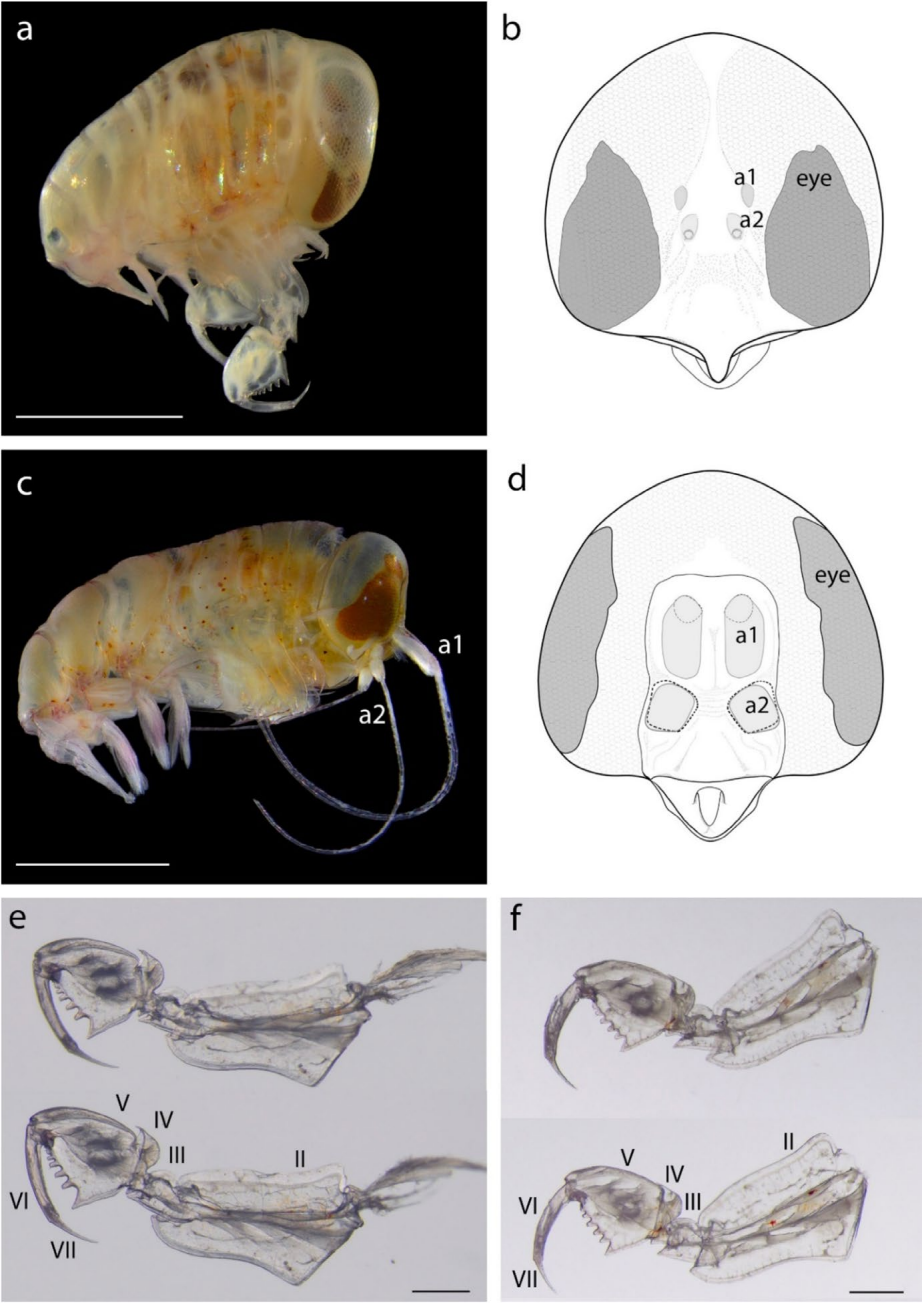
*Anchylomera blossevillei* . (a) female, side view; (b) female's head, frontal view; (c) male, side view; (d) male's head, frontal view. Light‐gray areas indicate the position of antennae 1 (a1) and antennae 2 (a2); (e) male's pereopod V. Lateral side (top) and medial side (bottom); (f) female's pereopod V. Lateral side (top) and medial side (bottom). Roman numerals indicate pereopod segments. Scale bar: (a) 2 mm; (c) 2 mm; (e, f) 500 μm.

Body length was 5.16 ± 0.68 mm in females (*n* = 124) and 4.84 ± 0.34 mm in males (*n* = 119). Head rounded, without rostrum, presenting sexual dimorphism. In females, the frontal side of the head has a slight indentation between the eyes; antennae 1 is very short, papillae‐like, and antennae 2 is reduced to a tubercle (Figure 4a,b). In males, the frontal side of the head has a distinct notch where the basal segments of the antennae are folded; antennae 1 and 2 are very long and exceed the body length (Figure 4c,d). Pereonites 1 and 2 are fused. Pereopods 1–2 are small. Pereopods 3–5 are subchelate. Pereopods 5 are massive with broad bases (II), having the greatest width proximally; the ischium (III) is small; the merus (IV) is small, scoop‐shaped; the carpus (V) is wide and well‐developed, with rounded teeth; the most proximal tooth is triangular in both sexes (Figure 4e,f); the propodus (VI) is thin and elongated with a sharp dactylus (VII). The urosome is shorter than the pleon. The uropods are rounded, with smooth margins. The telson is round‐triangular, nearly as wide as the urosome. No gregarines or ciliates were found in the body cavities of the specimens collected (*n* = 40).

### 3.3 Sex Ratio

The male:female ratio in the sample of 2024 was 0.99 (*n* = 313). The specimens in the sample of 2023 were in a progressively decomposed condition with most broken antennae and damaged heads, hampering a correct sex determination. All specimens were adults, and no gravid or brooding females were found.

### 3.4 Molecular Analysis

**FIGURE 5:**
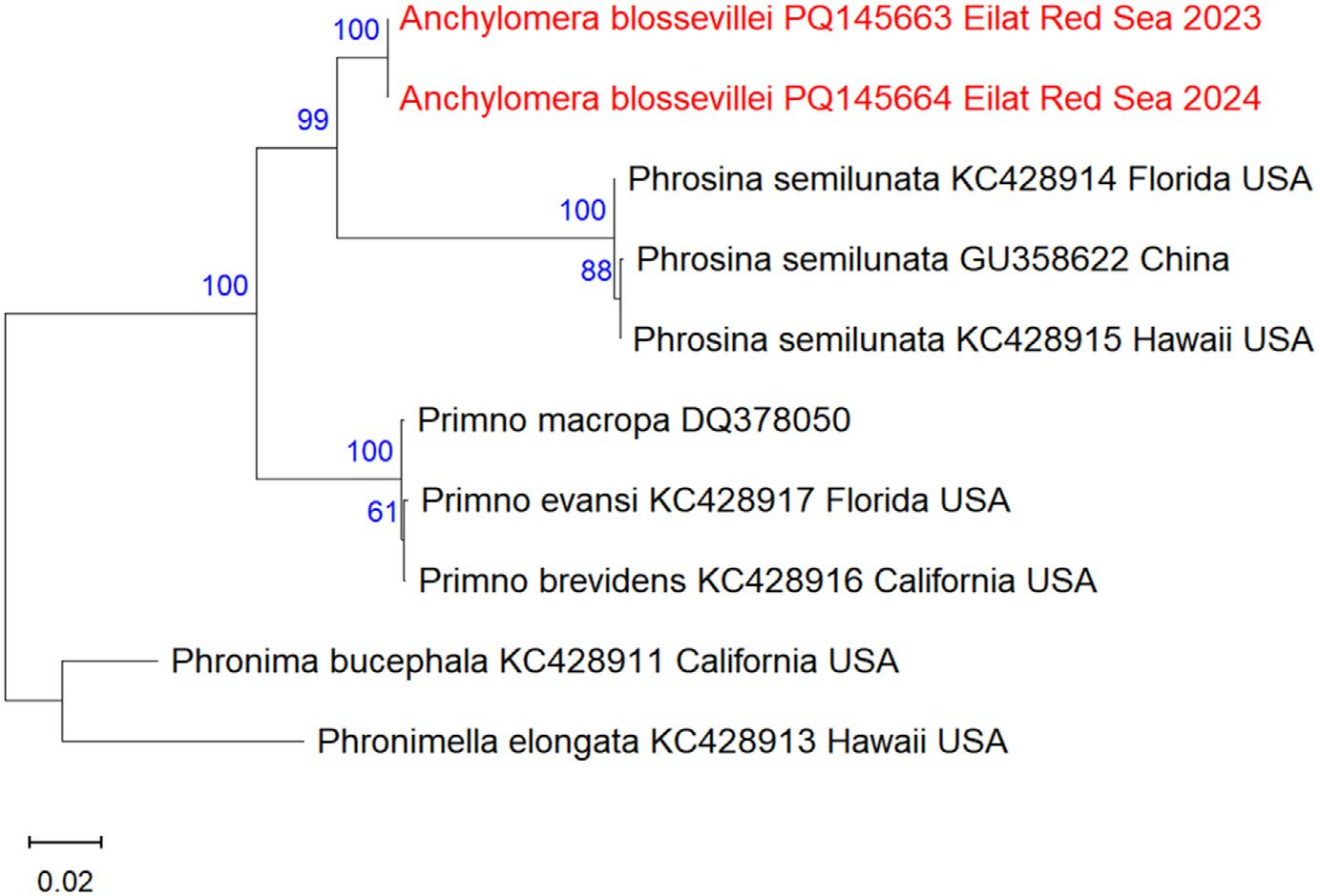
Maximum‐Likelihood phylogenetic tree of *Anchylomera blossevillei* based on the 18S rRNA gene, using the K2 + G substitution model. The outgroups *Phronima bucephala* and *Phronimella elongata* were used as a root node. The numbers in blue indicate the percentage of ML bootstrap support (1000 replicates) for nodes that received at least 60% support. The scale bar denotes the estimated number of nucleotide substitutions per site.

A DNA fragment of 631–671 bp of the 18S rRNA gene was sequenced from two thawed specimens collected in Electric Company Beach, Eilat in March 2023, and North Beach, Eilat in April 2024. The sequences were deposited in NCBI GenBank (https://www.ncbi.nlm.nih.gov/genbank/) under the accession numbers PQ145663 (2023) and PQ145664 (2024). Maximum likelihood analysis of 18S rRNA sequences obtained from GenBank showed that the two specimens from the Red Sea created a distinct cluster within the Phrosinidae family, forming a sister taxon to *Phrosina semilunata* and to the three *Primno* species, with high bootstrap support (Figure 5). As no previous sequences of *Anchylomera blossevillei* are currently available in NCBI GenBank and BOLD Systems (https://www.boldsystems.org/), the identity of *A. blossevillei* can be inferred from its phylogenetic position.

### 3.5 Literature Review of Hyperiid Mass Mortality Events

The results of the literature and citizen science repository review are detailed in Table 1 and illustrated in Figure 1. Selected photographs of hyperiid beach stranding events were added to Figure 2. Overall, 63 hyperiid swarming and die‐off events were found. The most abundant events were of the genus *Themisto* (*n* = 42), while seven die‐off events were recoded for *A. blossevillei* . Other documented stranding events were of *Oxycephalus clausi* (*n* = 3), *Phronima* sp. (*n* = 2), *Hyperia* spp. (*n* = 2), *Brachyscelus* sp. (*n* = 1), *Cyclocaris guilelmi* (*n* = 1), and other non‐identified hyperiids (*n* = 5). The regions where the swarming and stranding events were documented were the South Pacific Ocean (*n* = 31), Arctic Ocean (*n* = 12), North Atlantic Ocean (*n* = 9), Indian Ocean (*n* = 5), South Atlantic Ocean (*n* = 3), North Pacific Ocean (*n* = 2), and Antarctic Ocean (*n* = 1). The majority of these sites were located in high latitudes (mean latitude 51.93° N and 43.18° S in the northern and southern hemispheres, respectively). The highest frequency of the die‐off events occurred during summer months in both the northern (June–July) and southern (November–December) hemispheres (Figure 1b).

**TABLE 1:** Worldwide swarming and mass stranding reports of hyperiid amphipods based on literature search (ISI Web Of Science) and iNaturalist reports on https://www.inaturalist.org/observations/ (accessed on 4 August 2024).

	Taxon	Lat	Lon	Site	Marine province	Date	Reference
1	*Anchylomera blossevillei*	29.55	34.95	Eilat, north Gulf of Aqaba, Red Sea	Indian Ocean	17/03/2023	This study
2	*Anchylomera blossevillei*	29.55	34.97	Eilat, north Gulf of Aqaba, Red Sea	Indian Ocean	22/04/2023	This study
3	*Anchylomera blossevillei*	29.55	34.97	Eilat, north Gulf of Aqaba, Red Sea	Indian Ocean	23/04/2024	This study
4	*Anchylomera blossevillei*	29.46	34.98	Aqaba, north Gulf of Aqaba, Red Sea	Indian Ocean	13/03/1977	Post and Svoboda (1980)
5	*Anchylomera blossevillei*	19.56	−155.97	Keauhou Bay, Hawaii	North Pacific Ocean	10/08/1982	Lobel and Randall (1986)
6	*Brachyscelus* sp.	5.56	−87.05	Isla Manuelita, Cocos Islands, Costa Rica	South Pacific Ocean	02/2002	Whitney and Motta (2008)
7	*Themisto gaudichaudii*	−34.19	18.43	False Bay, South Africa	South Atlantic Ocean	20/01/2022	Brown and Gibbons (2022)
8	*Themisto libellula*	78.22	15.63	Adventfjorden, Svalbard	Arctic Ocean	10/09/2000	Eiane and Daase (2002)
9	*Themisto gaudichaudii*	54.44	−0.53	Robin Hood's Bay, Yorkshire, UK, North Sea	North Atlantic Ocean	26/06/1966	Gray and McHardy (1967)
10	*Themisto compressa*	54.62	−1.04	Redcar, Yorkshire, UK, North Sea	North Atlantic Ocean	04/1907	Gray and McHardy (1967)
11	*Themisto compressa*	42.17	−68.66	Georges bank, Gulf of Maine	North Atlantic Ocean	20/09/1932	Fish and Johnson (1937)
12	*Themisto gaudichaudii*	47.16	−2.61	off the French Atlantic coast, Bay of Biscay	North Atlantic Ocean	1920	Le Danois (1921)
13	*Themisto gaudichaudii*	−64.00	153.00	Antarctic Ocean	Antarctic Ocean	1957	Nemoto (1957)
14	*Hyperietta luzoni*	−48.04	166.56	Snares Islands, NZ	South Pacific Ocean	30/11/1976	Fenwick (1978)
15	*Themisto gaudichaudii*	−48.04	166.56	Snares Islands, NZ	South Pacific Ocean	30/11/1976	Fenwick (1978)
16	*Themisto compressa*	−48.04	166.56	Snares Islands, NZ	South Pacific Ocean	30/11/1976	Fenwick (1978)
17	*Themisto libellula*	79.00	−5.00	Fram Strait, Greenland Sea	Arctic Ocean	#N/A	Havermans et al. (2019)
18	*Themisto abyssorum*	78.17	13.66	Svalbard Bank, Barents Sea	Arctic Ocean	1990–1997	Dalpadado (2002)
19	*Themisto libellula*	79.00	−5.00	Fram Strait, Greenland Sea	Arctic Ocean	06/08/2000	Auel and Werner (2003)
20	*Themisto japonica*	37.26	138.25	off Joetsu, Sea of Japan	North Pacific Ocean	15/10/1984	Hirota and Semura (1990)
21	*Themisto compressa*	48.00	−17.50	Northeast Atlantic Ocean	North Atlantic Ocean	05/1990	Lampitt et al. (1993)
22	*Themisto abyssorum*	72.02	14.73	Haakon Mosby Mud Volcano, Norwegian Sea	Arctic Ocean	05/07/1998	Vinogradov (1999b)
23	*Themisto abyssorum*	64.78	4.78	Voring Plateau, Norwegian Sea	Arctic Ocean	06/07/1998	Vinogradov (1999b)
24	*Themisto libellula*	79.12	6.17	Vestnesa Ridge, Greenland Sea	Arctic Ocean	24/07/1998	Vinogradov (1999b)
25	*Cyclocaris guilelmi*	74.00	−10.00	Fram Strait, Greenland Sea	Arctic Ocean	08/2007	Kraft et al. (2013)
26	*Anchylomera blossevillei*	−33.50	153.00	warm‐core eddy J, Tasman Sea	South Pacific Ocean	08/1979	Young and Anderson (1987)
27	*Anchylomera blossevillei*	2.95	73.50	Maldives	Indian Ocean	28/03/2018	iNaturalist: 10469402
28	*Themisto* sp.	65.60	−20.64	Iceland	Arctic Ocean	13/07/2024	iNaturalist: 232911530
29	Hyperiidae	47.63	−52.86	Newfoundland island, Canada	North Atlantic Ocean	21/05/2024	iNaturalist: 217389531
30	*Oxycephalus clausi*	−36.09	173.86	Northland, NZ, Tasman Sea	South Pacific Ocean	08/03/2024	iNaturalist: 201631784
31	*Phronima sp*.	−36.10	173.87	Northland, NZ, Tasman Sea	South Pacific Ocean	20/02/2024	iNaturalist: 199888003
32	*Themisto gaudichaudii*	−46.61	169.38	Northland, NZ, Tasman Sea	South Pacific Ocean	28/01/2024	iNaturalist: 197770522
33	*Themisto* sp.	41.82	−70.00	Massachusetts, USA	North Atlantic Ocean	09/10/2023	iNaturalist: 187723149
34	*Themisto libellula*	78.51	11.31	Prince Charles Foreland, Svalbard	Arctic Ocean	16/07/2020	iNaturalist: 187416721
35	*Themisto libellula*	69.25	−53.52	Disko Island, Greenland	Arctic Ocean	05/08/2023	iNaturalist: 178088743
36	*Themisto* sp.	−45.65	170.66	Otago, NZ, Tasman Sea	South Pacific Ocean	03/06/2023	iNaturalist: 165260893
37	*Themisto compressa*	43.93	−60.02	Nova Scotia, Canada	North Atlantic Ocean	01/06/2023	iNaturalist: 164961059
38	*Oxycephalus clausi*	−36.10	173.87	Northland, NZ, Tasman Sea	South Pacific Ocean	15/05/2023	iNaturalist: 161764713
39	*Oxycephalus clausi*	−36.11	174	Northland, NZ, Tasman Sea	South Pacific Ocean	07/01/2023	iNaturalist: 145999499
40	*Phronima* sp.	−36.10	173.87	Northland, NZ, Tasman Sea	South Pacific Ocean	06/01/2023	iNaturalist: 145933323
41	Hyperiidae	−36.10	173.87	Northland, NZ, Tasman Sea	South Pacific Ocean	06/01/2023	iNaturalist: 145931610
42	Hyperiidae	−46.55	169.47	Purakauiti 9586, NZ	South Pacific Ocean	16/12/2022	iNaturalist: 145785953
43	*Themisto* sp.	−46.55	169.47	Tahakopa River, Catlins, Otago, NZ	South Pacific Ocean	16/12/2022	iNaturalist: 145645837
44	*Themisto* sp.	−45.64	170.64	Te Waipounamu, Waikouaiti, NZ	South Pacific Ocean	26/12/2022	iNaturalist: 145093056
45	*Themisto gaudichaudii*	−46.56	169.48	Tahakopa Bay, Clutha, Otago, NZ	South Pacific Ocean	16/12/2022	iNaturalist: 144776709
46	*Themisto gaudichaudii*	−45.62	170.67	Kaunihera a‐Rohe o Ōtepoti, NZ	South Pacific Ocean	14/12/2022	iNaturalist: 144398923
47	*Themisto* sp.	−45.72	170.60	South Pacific Ocean, Otago, NZ	South Pacific Ocean	14/12/2022	iNaturalist: 144310205
48	*Themisto* sp.	−45.88	170.70	South Pacific Ocean, Otago, NZ	South Pacific Ocean	10/12/2022	iNaturalist: 144120639
49	*Themisto australis*	−45.62	170.67	Waikouaiti, NZ	South Pacific Ocean	30/04/2022	iNaturalist: 122418739
50	Hyperiidae	−34.20	18.47	Cape Town, South Africa	South Atlantic Ocean	14/05/2022	iNaturalist: 117280266
51	*Themisto* sp.	−45.94	170.36	Ocean View, Dunedin, NZ	South Pacific Ocean	24/04/2022	iNaturalist: 115254784
52	*Themisto* sp.	−45.93	170.41	Kaunihera a‐Rohe o Ōtepoti, NZ	South Pacific Ocean	31/03/2022	iNaturalist: 110073820
53	*Themisto* sp.	−34.14	18.43	Fish Hoek, Cape town, South Africa	South Atlantic Ocean	31/01/2022	iNaturalist: 105893993
54	*Themisto* sp.	−45.91	170.54	Ocean Grove, Dunedin, NZ	South Pacific Ocean	09/12/2021	iNaturalist: 102806951
55	*Themisto australis*	−45.93	170.41	Dunedin, Otago, NZ	South Pacific Ocean	02/05/2020	iNaturalist: 44556972
56	*Hyperia galba*	43.93	−60.01	Sable Island, California, USA	North Atlantic Ocean	24/06/2021	iNaturalist: 84631976
57	*Themisto australis*	−45.91	170.51	Lawyers Head, Dunedin, NZ	South Pacific Ocean	01/01/2019	iNaturalist: 84286186
58	*Themisto* sp.	−46.47	169.76	South Otago, Cannibal Bay, NZ	South Pacific Ocean	07/04/2021	iNaturalist: 79114060
59	*Themisto* sp.	−45.93	170.42	Otago, NZ	South Pacific Ocean	17/12/2020	iNaturalist: 66727101
60	*Themisto australis*	41.10	176.07	Riversdale, NZ	South Pacific Ocean	26/12/2019	iNaturalist: 37417915
61	Hyperiidae	−40.61	176.41	Akitio, NZ	South Pacific Ocean	01/12/2019	iNaturalist: 36244308
62	*Themisto libellula*	69.57	−138.92	Beaufort Sea, Canada	Arctic Ocean	23/06/2016	iNaturalist: 11551112
63	*Themisto* sp.	−41.33	174.80	Lyall Bay, NZ	South Pacific Ocean	04/12/2015	iNaturalist: 2451015

*Note:* Latitude and longitude are presented in decimal degrees.

## 4 Discussion

Beach stranding *en masse* of marine organisms has long caught people's attention, dating back to ancient times, with historical references found in classical texts and folklore (e.g., Aristotle, ca. 350 BC). Often, these occurrences result from mass mortality of swarming marine organisms induced by a variety of biotic and abiotic environmental factors (Brusius, de Souza, and Barbieri 2020). Swarming and stranding of hyperiid amphipods, an abundant suborder of pelagic plankters, have been vastly documented in the scientific literature and by citizen scientists (Table 1). While many hyperiid species are known to be associated with gelatinous plankton as parasites or commensals (Harbison, Biggs, and Madin 1977; Laval 1980; Phleger et al. 1999; de Lima and Valentin 2001; Gasca and Haddock 2004; Gasca, Hoover, and Haddock 2015), the swarming and stranding reports primarily include free‐living species. A few hyperiids that are known to be associated with gelatinous plankton (e.g., *Phronima* sp., *Hyperia galba* ), were found stranded together with their hosts (e.g., salps, siponophores, scyphomedusae). Yet, the majority of the stranding events were of the free‐living hyperiid genus *Themisto*, predominately occurring in higher latitudes in the Arctic and the South Pacific oceans (Figure 1).

Tornado‐shaped swarms of *Themisto gaudichaudii* have been observed in shallow waters (2–3 m) along the South Atlantic coast of False Bay, South Africa (Brown and Gibbons 2022). Following this observation, the amphipod swarms stranded along the shores “in strings”, extending hundreds of meters in length. In the Norwegian and Greenland Seas, swarms of *Themisto* blocking the engine water‐intake pipes forced ships to stop (Vinogradov 1999a). Gray and McHardy (1967) reported instances (referred to as “invasions”) of the swarming and stranding of *T. gaudichaudii* along the Yorkshire coastline in the UK, with records dating back to 1892. While re‐examining the specimens in these events, they noted that the hyperiids were in reproductive state. Similar reports of gravid females in beach stranding (Dunbar 1957; Wing 1976) suggest that semelparity—a single reproductive lifespan followed by death—was the driving factor behind these die‐off events. The growth‐reproduction trade‐off and parental care have been linked to semelparous reproduction (Varpe and Ejsmond 2018). Semelparous amphipods were mainly associated with habitats that have a pronounced seasonality of food supply, such as the polar regions (Leonardsson, Sörlin, and Samberg 1988; Klages 1993; Ishikawa, Narita, and Urabe 2004), however, some warm temperate species can display semelparity under conducive environmental conditions (Sudo and Azeta 1996; Varpe and Ejsmond 2018).

Here, we report consecutive spring mass mortality and stranding events of the hyperiid amphipod *Anchylomera blossevillei* H. Milne Edwards, 1830 (Phrosinidae) in the northern Gulf of Aqaba, Red Sea. This monotypic species has a widespread cosmopolitan distribution, predominantly occurring in tropical and temperate regions (Zeidler 2004). Risso (1826) recorded *A. blossevillei* as an associate of chordate pyrosomes, and Harbison, Biggs, and Madin (1977) recorded it preying on the siphonophore *Forskalia tholoides* ; however, its association with gelatinous plankton has not been confirmed and is considered unlikely. In the northern Gulf of Aqaba, *A. blossevillei* was first described by Ruffo (1958) and Furnestin (1958), and later by Echelman and Fishelson (1990) as a common, yet not dominant, member of the pelagic zooplankton community. The first mass die‐off event of *A. blossevillei* in the northern Gulf of Aqaba was documented in March 1977 by Post and Svoboda (1980), who found “millions of specimens… washed ashore… the beach in front of the marine station had turned purple.”. Lobel and Randall (1986) described dense, tornado‐like swarms of *A. blossevillei* off the Kona coast, Hawaii, and linked this phenomenon with the influence of a co‐occurring cold‐core cyclonic eddy. They reported a sex ratio of 0.42 (male:female), with 19% of the females being gravid and 3% brooding, which thus does not support semelparity. We found a male:female ratio of 0.99 in *A. blossevillei* in the stranding events at the northern Gulf of Aqaba, significantly higher than in swarms of semelparous hyperiids (0.08–0.37, Siegfried 1965; Brown and Gibbons 2022). Based on this evidence and the lack of gravid or brooding females, we can reject the semelparous reproduction hypothesis.

Marine heatwaves (MHWs)—extended periods of anomalously warm ocean temperatures—are becoming increasingly common (Oliver et al. 2018), often leading to catastrophic consequences for marine communities (Hoegh‐Guldberg et al. 2007; Smale et al. 2019). While most of the detrimental impacts of MHWs have been recorded in benthic communities (Giraldo‐Ospina, Kendrick, and Hovey 2020; Garrabou et al. 2022; Smith et al. 2023), mass mortalities of pelagic fish and megafauna have been documented (Cavole et al. 2016; Wild et al. 2019; Trainer et al. 2020), facilitated by deoxygenation or harmful algal blooms. In the northern Gulf of Aqaba, a mass mortality of over 40 species of reef fish was recorded between June and August 2017, during a MHW event with an extreme increase of 4.2°C in 2.5 days (Genin et al. 2020). In addition to the thermal stress induced by that event, the fish became fatally infected with *Streptococcus iniae* , a common Red Sea bacterial pathogen. Previous studies of pathogenic infections in amphipods showed impacts on reproduction rather than survival (Ohtsuka et al. 2004; Bojko and Ovcharenko 2019). Nonetheless, Prokopowicz et al. (2010) hypothesized that gregarine and ciliate parasites found in the hyperiid *T. libellula* in high densities may have killed their hosts. In our study, we have not found evidence for gregarine or ciliate parasites in the stranded *A. blossevillei* . Temperatures before and during the stranding events exhibited variations within the seasonal amplitude (Silverman and Gildor 2008; Sengupta, Gildor, and Ashkenazy 2024). Thus, we can infer that the mass die‐offs of *A. blossevillei* in the Gulf of Aqaba were not caused by thermal stress or parasitic infection.

In March 1977, among the millions of stranded *A. blossevillei* in Aqaba, Jordan, Post and Svoboda (1980) found scattered decapods, stomatopods, cephalopods, and mesopelagic fish. Although the authors initially linked the stranding to an underwater detonation that occurred a day earlier, they suggested that the presence of mesopelagic fish among the stranded organisms indicates that the event was caused by strong upwelling in the narrow‐shelf Gulf of Aqaba. Previous studies have linked hyperiid swarming and mass die‐off events with mesoscale eddies—closed circular currents, typically 10 to 200 km in diameter (Shulenberger 1978; Young and Anderson 1987; Young 1989; Gasca 2003, 2004; Lavaniegos and Hereu 2009). Eddies form partially isolated environments with distinct physical and chemical conditions, supporting and transporting whole plankton communities (Owen 1981; Olson 1991; Condie and Condie 2016). The high concentrations of hyperiids in mesoscale structures were mainly explained by advection from offshore waters (Young 1989; Lavaniegos and Hereu 2009). Young and Anderson (1987) and McWilliam and Phillips (1983) found swarms of *A. blossevillei* at the cooler surface waters outside a warm‐core eddy in the Tasman Sea. Similarly, an analysis of the hyperiid amphipod assemblages from the Gulf of Ulloa, Baja California, showed high abundances of *A. blossevillei* during summertime, characterized by coastal upwelling events (Lavaniegos 2020). Seasonal coherent cyclonic eddies have been recorded in the northern part of the Gulf of Aqaba, Eilat, using a high‐frequency radar system (Gildor, Fredj, and Kostinski 2010). These surface eddies, characterized by a basin‐wide diameter of 5–6 km, prevail from November to April and typically persist for around a day. Under weak wind conditions, as measured in this study, an eastward current at the southern open boundary of the rectangular‐shaped area of the head of the gulf drives a cavity‐flow cyclonic circulation. The eddy currents are stronger than the typical current velocity of 10–20 cm s^−1^ and can exceed 100 cm s^−1^ (Afargan and Gildor 2015). Such cyclonic eddies may have facilitated the stranding events of the hyperiid *A. blossevillei* in the Gulf of Aqaba during March 1977 (Post and Svoboda 1980) and March and April 2023–2024.

Swarming hyperiids are important prey in many pelagic food webs and a major link from mesozooplankton secondary production to higher trophic levels such as seabirds and marine mammals (Auel and Werner 2003). In the Arctic, *T. libellula* represents a significant and stable food resource for polar cods (Lønne and Gabrielsen 1992), seabirds (Węsławski et al. 1999; Pedersen and Falk 2001), and seals (Wathne, Haug, and Lydersen 2000). The Sei whale in the Antarctic has been reported to feed almost exclusively on *T. gaudichaudii* , while the longfin tuna is known to feed on immense surface swarms of this species (Gray and McHardy 1967). In the Mediterranean Sea, stomach content analyses revealed that *A. blossevillei* is an essential prey item of the short‐finned squid *Illex coindetii* (Martínez‐Baena et al. 2016), the little tunny *Euthynnus alletteratus* (Falautano et al. 2007), and the bullet tuna *Auxis rochei* (Mostarda et al. 2007). In the northern Gulf of Aqaba, Red Sea, coral‐inhabiting *Trapezia* crabs have been shown to extensively feed on hyperiids (Shmuel, Ziv, and Rinkevich 2022). However, more data is required to determine the role of *A. blossevillei* in the Red Sea food webs. Further impacts of mass stranding include the facilitation of coastal cycling and remineralization of carbon, nitrogen, and phosphorus via microbial decomposition of the animal carcasses (Schlacher et al. 2020). In oligotrophic ecosystems, where allochthonous inputs are scant, the importance of such autochthonous sources is high (Guy‐Haim et al. 2020). Future research is needed to assess the potential impacts of the globally prevalent hyperiid mass die‐off events on marine ecosystems.

## Author Contributions

**Tamar Guy‐Haim:** conceptualization (lead), data curation (lead), formal analysis (lead), funding acquisition (lead), investigation (lead), resources (lead), visualization (lead), writing – original draft (lead), writing – review and editing (equal). **Anastasiia Iakovleva:** formal analysis (supporting), investigation (supporting), methodology (supporting), visualization (supporting), writing – review and editing (supporting). **Viviana Farstey:** conceptualization (supporting), investigation (supporting), validation (lead), writing – review and editing (supporting). **Ayah Lazar:** validation (supporting), writing – review and editing (supporting). **Khristina Ermak:** visualization (supporting), writing – review and editing (supporting). **Arseniy R. Morov:** formal analysis (supporting), investigation (supporting), methodology (lead), validation (supporting), visualization (supporting), writing – review and editing (supporting).

## Conflicts of Interest

The authors declare no conflicts of interest.

## Data Availability

The molecular data presented in this article are available in the GenBank Nucleotide Database at https://www.ncbi.nlm.nih.gov/genbank/, and can be accessed with accession numbers PQ145663.1 and PQ145664.1.
